# Supplementary material for: Investigation on the Combined Effect of Hydroxypropyl Beta-Cyclodextrin (HPβCD) and Polysorbate in Monoclonal Antibody Formulation
Source: Pharmaceuticals (Basel). 2024 Apr 19;17(4):528. doi: 10.3390/ph17040528 (PMC11054243; doi:10.3390/ph17040528)
Supplement: Supplementary file 1 [file pharmaceuticals-17-00528-s001.zip › pharmaceuticals-2947044-supplementary.pdf]

Supplementary data

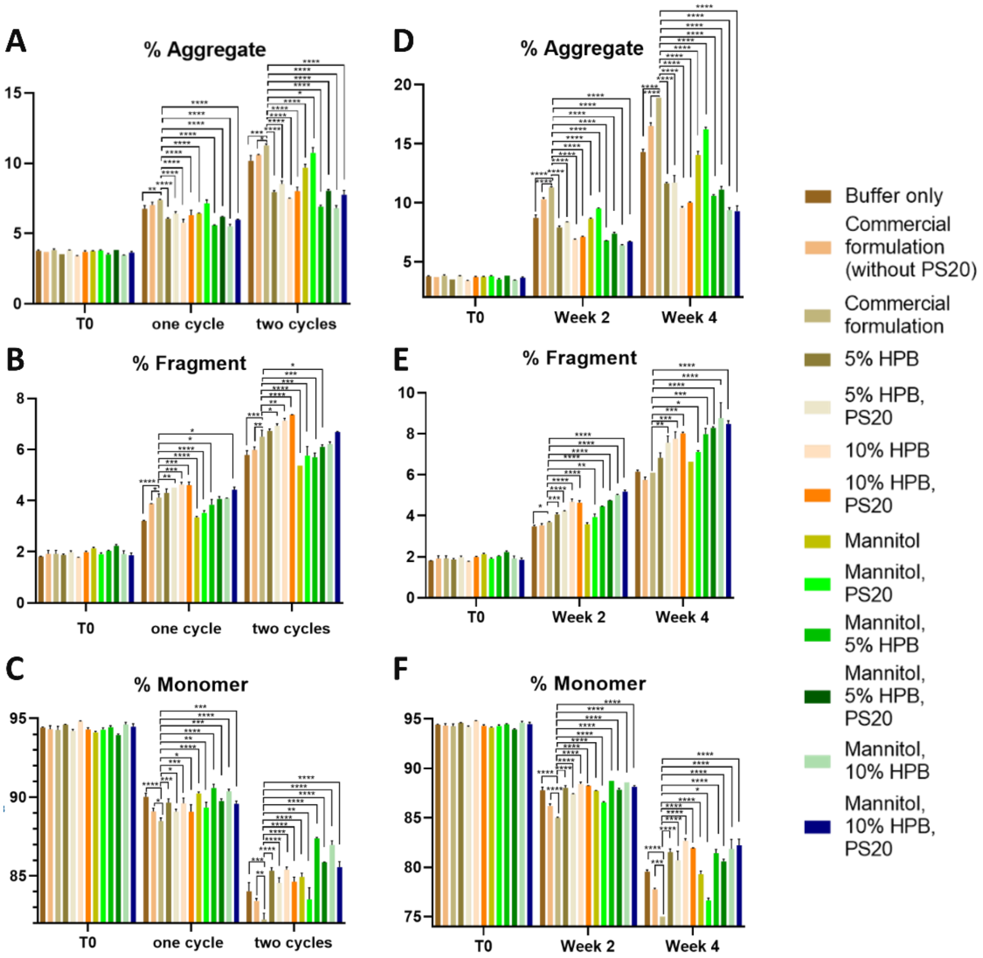

Supplementary Figure S1. Bevacizumab size profiling under light and heat stresses. Aggregation (A), fragmentation (B) and monomer recovery (C) of bevacizumab in different formulations under light stress for one and two cycles. Aggregation (D), fragmentation (E) and monomer recovery (F) of bevacizumab in different formulations under heat stress for two and four weeks. * $p < 0.05$, ** $p < 0.01$, *** $p < 0.001$, and **** $p < 0.0001$ using one-way ANOVA compared to the commercial formulation.

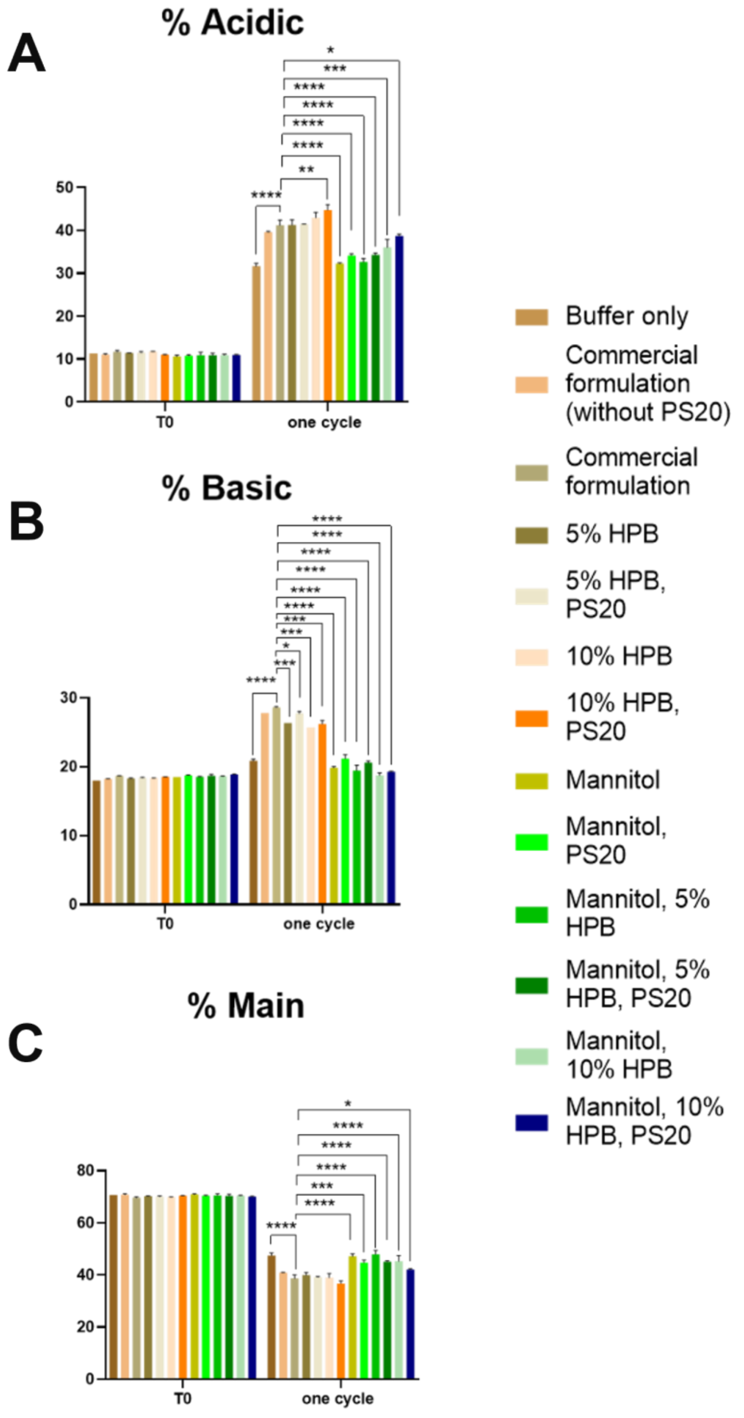

Supplementary Figure S2. Bevacizumab charge variants profiling under light stress. Acidic variants (**A**), basic variants (**B**) and main peaks (**C**) of bevacizumab in different formulations under light stress. * $p < 0.05$, ** $p < 0.01$, *** $p < 0.001$, and **** $p < 0.0001$ using one-way ANOVA compared to the commercial formulation.

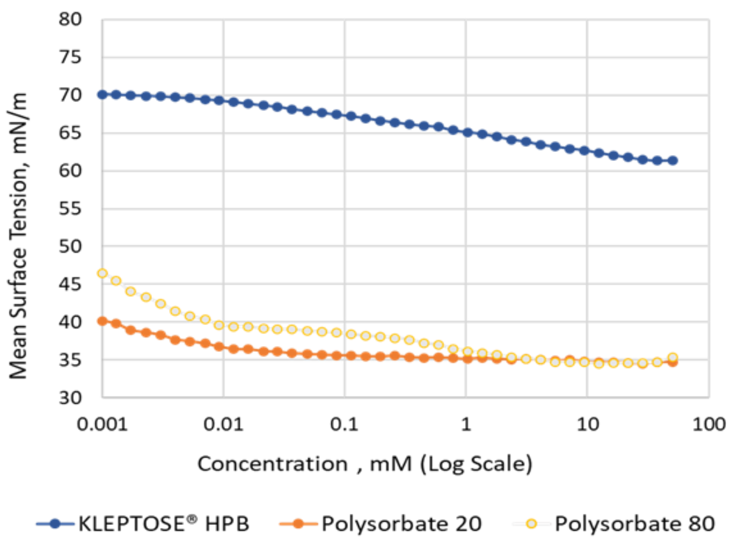

Supplementary Figure S3. Static or equilibrium surface tension of KLEPTOSE® HPB, polysorbate 20 and polysorbate 80.

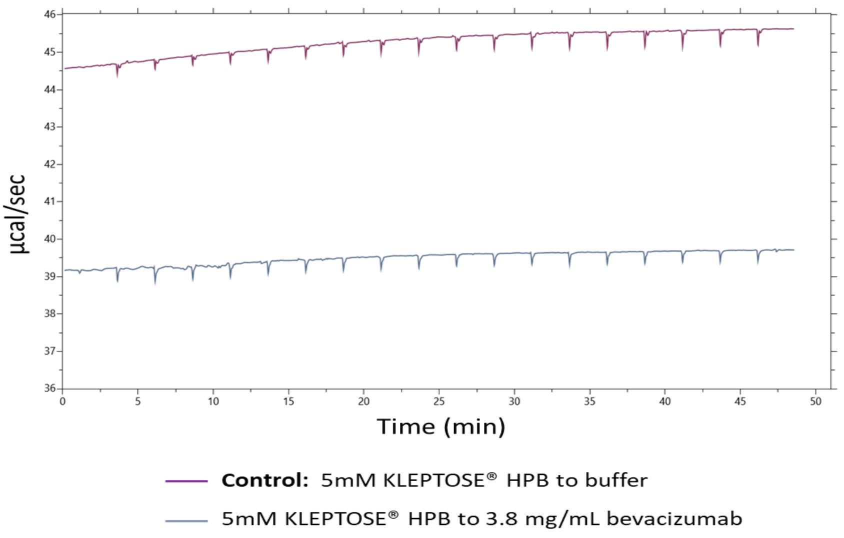

Supplementary Figure S4. Isothermal titration calorimetry analysis of KLEPTOSE® HPB and bevacizumab. Blue curve: Titrating 5 mM KLEPTOSE® HPB to 3.8 mg/mL bevacizumab in buffer. Magenta: Titrating 5 mM KLEPTOSE® HPB to buffer.
